# Silencing GGH induces autophagy by increasing folate stress and production of NADH

**DOI:** 10.1093/jmcb/mjaf014

**Published:** 2025-04-23

**Authors:** Yu Li, Yuhui Du, Sijie Chen, Zhangrong Xie, Xinrui Li, Baoyue Lin, Zhiqing Zhou, Huijie Zhao, Guoan Chen

**Affiliations:** Department of Human Cell Biology and Genetics, Joint Laboratory of Guangdong–Hong Kong Universities for Vascular Homeostasis and Diseases, School of Medicine, Southern University of Science and Technology, Shenzhen 518055, China; Department of Human Cell Biology and Genetics, Joint Laboratory of Guangdong–Hong Kong Universities for Vascular Homeostasis and Diseases, School of Medicine, Southern University of Science and Technology, Shenzhen 518055, China; Department of Human Cell Biology and Genetics, Joint Laboratory of Guangdong–Hong Kong Universities for Vascular Homeostasis and Diseases, School of Medicine, Southern University of Science and Technology, Shenzhen 518055, China; Department of Human Cell Biology and Genetics, Joint Laboratory of Guangdong–Hong Kong Universities for Vascular Homeostasis and Diseases, School of Medicine, Southern University of Science and Technology, Shenzhen 518055, China; Department of Human Cell Biology and Genetics, Joint Laboratory of Guangdong–Hong Kong Universities for Vascular Homeostasis and Diseases, School of Medicine, Southern University of Science and Technology, Shenzhen 518055, China; Department of Human Cell Biology and Genetics, Joint Laboratory of Guangdong–Hong Kong Universities for Vascular Homeostasis and Diseases, School of Medicine, Southern University of Science and Technology, Shenzhen 518055, China; Department of Critical Care Medicine, The First Affiliated Hospital, Zhejiang University School of Medicine, Hangzhou 311399, China; Department of Human Cell Biology and Genetics, Joint Laboratory of Guangdong–Hong Kong Universities for Vascular Homeostasis and Diseases, School of Medicine, Southern University of Science and Technology, Shenzhen 518055, China; Department of Human Cell Biology and Genetics, Joint Laboratory of Guangdong–Hong Kong Universities for Vascular Homeostasis and Diseases, School of Medicine, Southern University of Science and Technology, Shenzhen 518055, China; Department of Oncology, Sun Yat-sen Memorial Hospital, Sun Yat-sen University, Guangzhou 510120, China; Department of Human Cell Biology and Genetics, Joint Laboratory of Guangdong–Hong Kong Universities for Vascular Homeostasis and Diseases, School of Medicine, Southern University of Science and Technology, Shenzhen 518055, China; The First Affiliated Hospital of Southern University of Science and Technology, Shenzhen 518055, China; SUSTech Homeostatic Medicine Institute, School of Medicine, Southern University of Science and Technology, Shenzhen 518055, China

**Keywords:** lung cancer, GGH, autophagy, FPGS, AMPK

## Abstract

There is an inextricable link between metabolic disorders and autophagy. Gamma-glutamyl hydrolase (GGH) is a lysosomal glycoprotein that reduces intracellular folate stress by catalyzing the hydrolysis of polyglutamylated folate into transportable monoglutamate. The relationship between folate metabolism, involving the folate metabolic enzyme GGH, and autophagy has rarely been reported. In this study, we found that GGH functions as a crucial oncogene in lung adenocarcinomas. Importantly, we found that cell autophagy and autophagic cell death are induced by GGH silencing through the elevated folate stress resulting from folate metabolism and the folate metabolite nicotinamide adenine dinucleotide (NADH). By increasing the NADH/NAD^+^ ratio, silencing GGH activates adenosine monophosphate-activated protein kinase (AMPK) through the activation of LKB1 and CAMKK2, as well as enhanced AMP/ATP and ADP/ATP ratios, which then triggers the initiation of early autophagy, finally resulting in autophagic cell death. Taken together, our study suggests that GGH may not only serve as a prognostic marker but also play a critical role in the initiation of early autophagy. Interventions targeting GGH to regulate folate metabolism and the proportion of NADH/NAD^+^ may have translational potential for precision therapy in human cancer.

## Introduction

Autophagy is a process of degrading cytoplasmic components, essential in cellular homeostasis and survival. Macroautophagy, the most prevalent form of autophagy, is regulated by autophagy-related genes. Autophagy proceeds through four steps: initiation of autophagy, phagophore nucleation, phagophore expansion, and fusion of autophagosome and lysosome leading to degradation (Dower et al., 2018). Initially, mechanistic target of rapamycine kinase (mTOR) and adenosine monophosphate-activated protein kinase (AMPK) respond to changes in nutritional conditions and upstream regulators and then activate the Unc-51-like autophagy activating kinase 1 (ULK1) kinase complex, which initiates autophagosome biogenesis at the phagophore assembly site (PAS) (Zachari and Ganley, 2017). Following this, Class III phosphatidylinositol 3-kinase (PI3KC3) complex I (PI3KC3-C1) induces nucleation of the phagophore, forming a bag-shaped isolation membrane (Axe et al., 2008). A range of autophagy-related proteins, such as ATG, facilitate the maturation of autophagosomes, which ultimately fuse with lysosomes for degradation (Russell et al., 2013). During this process, the conversion of LC3-I to LC3-II serves as an important indicator for assessing autophagy activity.

Autophagy maintains cellular homeostasis during low-energy states. Several substances work as energy molecules to sustain cell growth, including ATP and nicotinamide adenine dinucleotide (NAD) (Rigoulet et al., 2020). A decrease in the NAD^+^/NADH ratio induces autophagy through multiple mechanisms (Zhang et al., 2016; Wilson et al., 2023). NAD^+^/NADH ratio exerts an influence on an array of upstream autophagic signaling cascades, including ATP generation, reactive oxygen species (ROS) production, and Ca^2+^ homeostasis, through various metabolic pathways such as glycolysis, the tricarboxylic acid (TCA) cycle, the electron transport chain (ETC), fatty acid β-oxidation, and IP3R signaling (Zhang et al., 2016).

Gamma-glutamyl hydrolase (GGH) is a highly conserved and ubiquitously expressed lysosomal glycoprotein that catalyzes the hydrolysis of polyglutamylated folate into transportable monoglutamate, thereby reducing the concentration of polyglutamylated folate in the cell and participating in folate metabolism (Schneider and Ryan, 2006). In addition, GGH is a known oncogene. Studies have shown that GGH is upregulated in multiple cancer types and associated with the phenotype and poor prognosis of some of these tumors (He et al., 2004; Kawakami et al., 2008; Wang et al., 2018). However, its oncogenic roles and underlying molecular mechanisms have not been fully studied. The relationship between folate metabolism, particularly folate metabolic enzymes like GGH, and macrophage autophagy has rarely been reported. This study focuses on how the folate hydrolase GGH regulates autophagy, exploring its oncogenic functions and providing a new perspective on the relationship between folate metabolism and macrophage autophagy.

## Results

### GGH has an essential oncogenic role in lung adenocarcinoma

**Figure 1:**
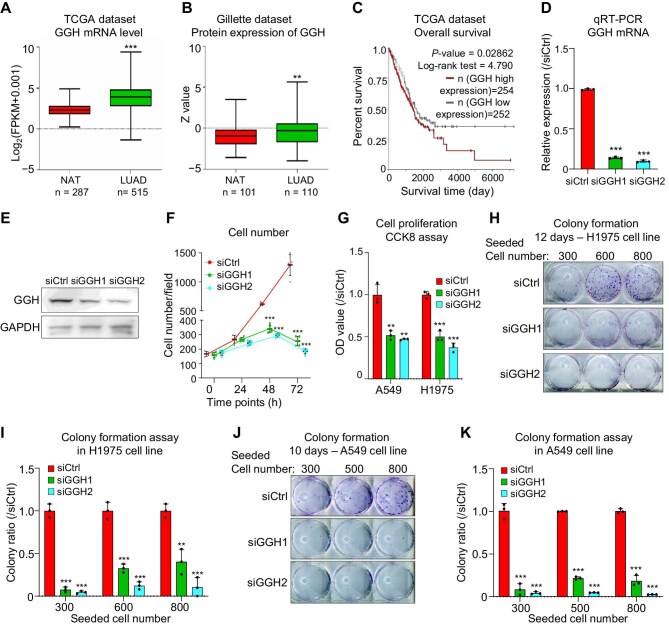
GGH has an essential oncogenic role in LUAD. (**A**) In TCGA dataset, the mRNA expression of GGH is significantly elevated in LUAD tissues compared to normal lung tissue (NAT). ***P < 0.001. (**B**) In the Gillette dataset, GGH protein expression is significantly increased in LUAD tissues compared to NAT. **P < 0.01. (**C**) Kaplan–Meier survival curves showing that high GGH mRNA expression is associated with poor prognosis in LUAD patients. (**D**) GGH mRNA expression was significantly reduced after siGGH1 or siGGH2 treatment in A549 cell line. (**E**) Western blot showing changes in GGH protein expression after siGGH1 or siGGH2 treatment in A549 cell line. (**F**) The effect of siGGH1 or siGGH2 on the cell number at different time points after transfection in A549 cell line. Data are shown as mean ± SD, *n* = 5. Five fields were randomly selected from each specimen under a microscope. ***P < 0.001 vs. siCtrl for the same time point. (**G**) Cell proliferation of A549 and H1975 cell lines after 48 h siGGH1 or siGGH2 treatment. Data are shown as mean ± SD, *n* = 3. **P < 0.01, ***P < 0.001. (**H** and **I**) Colony formation of H1975 cells cultured for 12 days after siGGH1 or siGGH2 transfection. Cells were seeded at 300, 600, and 800 cells/well in 6-well plates. Data are shown as mean ± SD, *n* = 3. **P < 0.01, ***P < 0.001. (**J** and **K**) Colony formation of A549 cells cultured for 10 days after siGGH1 or siGGH2 transfection. Cells were seeded at 300, 500, and 800 cells/well in 6-well plates. Data are shown as mean ± SD, *n* = 3. ***P < 0.001.

Although GGH has been reported to play varying roles in different cancer types, with potentially distinct biological functions, its role in lung adenocarcinoma (LUAD) remains unclear. In The Cancer Genome Atlas (TCGA) database, GGH mRNA expression levels in LUAD samples were significantly higher than those in normal lung tissue (*P *< 0.001) (Figure 1A; Cancer Genome Atlas Research Network, 2014). Similarly, in the Gillette dataset, GGH protein levels were significantly elevated in LUAD samples compared to normal lung tissue (*P *< 0.01) (Figure 1B; Gillette et al., 2020). In addition, LUAD patients with high levels of GGH mRNA showed lower overall survival rates than those with low GGH levels (*P *< 0.01) (Figure 1C).

To detect the role of GGH in human LUAD cells, knockdown experiments were performed. GGH siRNA knockdown efficiency at mRNA and protein levels was measured by quantitative real-time polymerase chain reaction (qRT-PCR) and western blot experiment, respectively, in A549 cells transfected with siRNAs (siCtrl, siGGH1, or siGGH2) (Figure 1D and E). Next, we conducted cell counting and statistical analysis in A549 cells and observed a significant reduction in cell number at both 48 h and 72 h following the treatment of siGGH1 and siGGH2 (Figure 1F; Supplementary Figure S1). By performing the CCK-8 assay, we showed that GGH silencing significantly reduced the proliferation of both A549 and H1975 cells (Figure 1G). After siRNA transfection, H1975 and A549 cells were cultured for 12 and 10 days, respectively. The following colony formation assays demonstrated that GGH knockdown significantly reduced the colony formation ability and induced cell death of A549 and H1975 cell lines (Figure 1H–K). These results prove that GGH has an essential oncogenic role in LUAD.

### GGH silencing induces autophagy

To explore whether GGH has a role in autophagy at the cellular level, we performed autophagy-related experiments after GGH inhibition. We found that silencing GGH could induce autophagy, a phenomenon that has not been reported previously.

**Figure 2:**
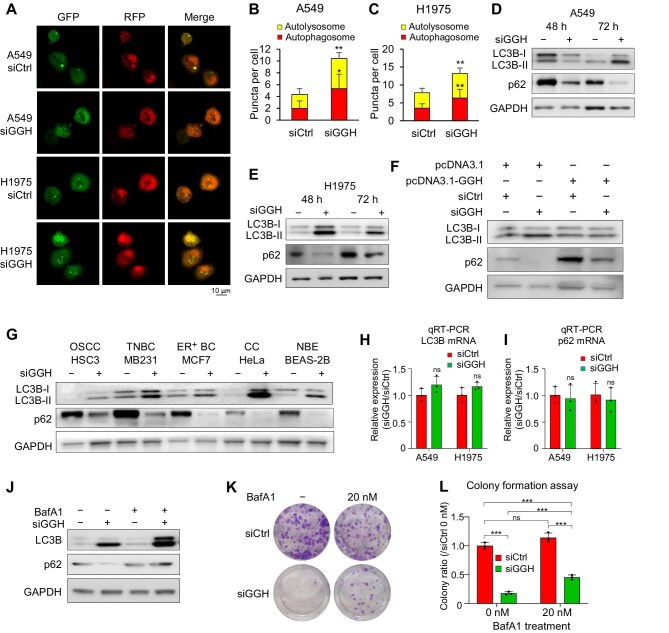
Silencing GGH induces autophagy. (**A**–**C**) A549 and H1975 cell lines were treated with siCtrl or siGGH for 24 h and infected with mRFP-GFP-LC3 tandem fluorescent protein adenovirus for 48 h. (**A**) Representative images showing autophagic flow. Images were captured using a fluorescence microscope at 400× magnification. Scale bar, 10 μm. (**B** and **C**) Quantitative analysis of autophagosomes (red puncta) and autolysosomes (yellow puncta). Data are shown as mean ± SD of triplicate culture wells (five images per well). (**D** and **E**) Western blot analysis of LC3B-I/LC3B-II and p62 proteins after 48 h and 72 h siGGH treatment in A549 and H1975 cell lines. (**F**) Western blot analysis of LC3B-I/LC3B-II and p62 proteins after siCtrl or siGGH treatment for 72 h, along with either a control (pcDNA3.1) or GGH overexpression (pcDNA3.1-GGH) plasmid for 48 h. (**G**) Western blot analysis of LC3B-I/LC3B-II and p62 proteins after 72 h siCtrl or siGGH treatment in diverse cancer cell lines, including HSC3 (OSCC, oral squamous cell carcinoma), MDA-MB-231 (TNBC, triple-negative breast cancer), MCF7 (ER^+^ BC, estrogen receptor-positive breast cancer), and HeLa (CC, cervical cancer), and normal bronchial epithelial (NBE) cell line BEAS-2B. (**H** and **I**) LC3B and p62 mRNA expression after siGGH treatment. Data are shown as mean ± SD, *n* = 3. (**J**) H1975 cells were transfected with siGGH for 72 h and treatment with 100 nM BafA1 for 6 h, followed by western blot analysis of LC3B and p62 proteins. (**K** and **L**) Colony formation of A549 cells cultured for 6 days (1000 cells/well) after treatment with siGGH and 20 nM BafA1 concurrently for 48 h. Data are shown as mean ± SD, *n* = 3. *P < 0.05, **P < 0.01, ***P < 0.001.

LC3B is a classical marker of autophagy, which is located in the membranes of autophagosomes, and plays a crucial role in autophagy induction (Li et al., 2022). Using the RFP-GFP-LC3B assay, where LC3B is tagged with acid-resistant RFP and/or acid-sensitive GFP to indicate the formation of autophagosomes (RFP-positive GFP-positive signal) and autolysosomes (low pH environment, RFP-positive GFP-negative signal) (Wang et al., 2018), we demonstrated that silencing GGH increased the formation of total autophagosomes and autolysosomes in A549 and H1975 cells (Figure 2A–C).

The conversion from non-lipidated form LC3B-I to lipidated form LC3B-II can serve as a marker for the occurrence of autophagy (Chen et al., 2020). The autophagy cargo receptor p62/SQSTM1 functions to bridge cargo and LC3B, and thus a lower p62 level is also a marker of autophagy (Campagnoli et al., 2022). We determined the protein levels of LC3B-I/LC3B-II and p62 at 48 h and 72 h after siGGH treatment by western blot analysis. After 48 h of siRNA interference, the upregulation of LC3B-I/II conversion and degradation of p62, indicating enhanced autophagosome synthesis and the initiation of protein degradation, were observed in H1975 cell cells, while these changes of the autophagy markers were still relatively weak in A549 cells (Figure 2D and E). After 72 h of siRNA interference, the upregulation of LC3B-I/II conversion and degradation of p62 were observed in both cell lines (Figure 2D and E). This suggests that GGH silencing can induce autophagy in both LUAD cell lines, although the timing of autophagy induction differs. Notably, these effects could be rescued by GGH overexpression (Figure 2F). Meanwhile, similar changes in LC3B and p62 protein levels were found in other types of cancer and normal lung cells after siGGH treatment (Figure 2G). The data-independent acquisition mass spectrometry (DIA-MS) and RNA sequencing (RNA-seq) results showed that while the total protein level of LC3B increased and that of p62 decreased, their mRNA levels had little change after siGGH interference (Figure 2H and I; Supplementary Figure S2). These results suggest that the changes in LC3B and p62 after siGGH intervention may occur at the protein level rather than the RNA level.

Subsequently, we employed the autophagy inhibitor bafilomycin A1 (BafA1), which inhibits the late stage of autophagy, in particular the fusion of autophagosomes and lysosomes. Following GGH knockdown, the BafA1 (100 nM)-treated group exhibited significantly higher levels of LC3B and p62 protein expression compared to the untreated group (Figure 2J). In addition, BafA1 (20 nM) intervention partially reversed cell death induced by siGGH treatment (Figure 2K and L). These results confirm that GGH knockdown can induce autophagy, leading to an increase in autophagic flux and finally cell death. When the final step of autophagy is blocked, autophagosomes cannot be degraded promptly, resulting in the accumulation of autophagy markers and inhibition of cell death.

### GGH knockdown promotes autophagy in the early steps

**Figure 3:**
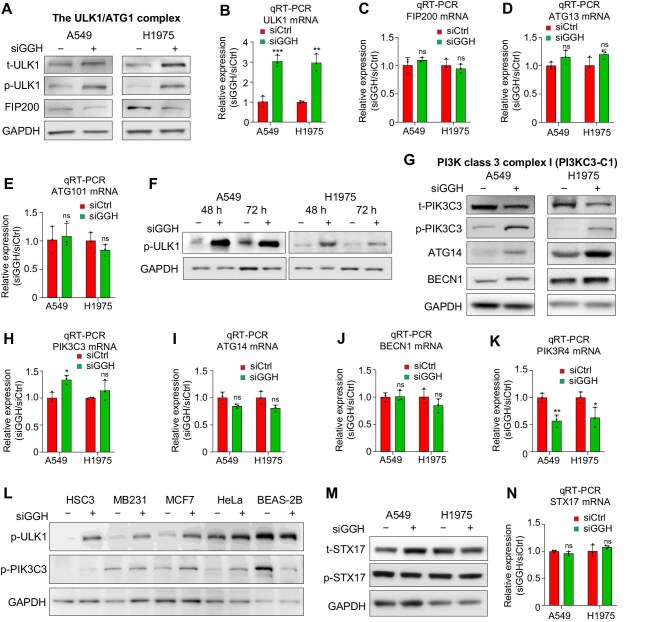
Silencing GGH induces early steps of autophagy. (**A**) Western blot analysis of t-ULK1, p-ULK1 (Ser555), and FIP200 after 72 h siGGH treatment. (**B**–**E**) mRNA expression levels of ULK1/ATG1 complex components. Data are shown as mean ± SD, *n* = 3. **P < 0.01, ***P < 0.001. (**F**) Western blot analysis of p-ULK1 (Ser555) after 48 h and 72 h siGGH treatment. (**G**) Western blot analysis of t-PIK3C3, p-PIK3C3 (Ser249), ATG14, and BECN1 after 72 h siGGH treatment. (**H**–**K**) mRNA expression levels of PI3KC3-C1 components. Data are shown as mean ± SD, *n* = 3. *P < 0.05, **P < 0.01. (**L**) Western blot analysis of p-ULK1 (Ser555) and p-PIK3C3 (Ser249) proteins after 72 h siCtrl or siGGH treatment in diverse cancer cell lines and NBE cell line. (**M**) Western blot analysis of t-STX17 and p-STX17 (Ser202) after 72 h siGGH treatment. (**N**) STX17 mRNA expression after siGGH treatment. Data are shown as mean ± SD, *n* = 3.

To further elucidate the role of GGH in autophagy, we examined multiple autophagy-related proteins at different stages upon GGH knockdown. During the first step, initiation of autophagy, the ULK1 complex is formed at the PAS, which consists of ULK1, ATG13, FIP200, and ATG101 (Licheva et al., 2022). ULK1 is central to this complex, as it can activate other components (Zachari and Ganley, 2017). Western blot and DIA-MS analyses showed the upregulation of total and Ser555-phosphorylated ULK1 protein, as well as ATG101 protein, following GGH knockdown (Figure 3A; Supplementary Figure S3A). qRT-PCR and RNA-seq results revealed that the mRNA level of ULK1 also increased (Figure 3B; Supplementary Figure S3B), while the mRNA expression of other components of the ULK1 complex did not change obviously (Figure 3C–E; Supplementary Figure S3C–E). We further determined the protein levels of Ser555-phosphorylated ULK1 protein at 48 h and 72 h after siGGH treatment. After 48 h of siRNA interference, the ULK1 protein was already activated (Figure 3F), suggesting that the occurrence of autophagy initiation predated 48 h after siGGH treatment in both LUAD cell lines.

The ULK1 complex regulates the recruitment of a second kinase complex, PI3KC3-C1, which is also called the PIK3C3/VPS34 complex 1. PI3KC3-C1 is responsible for the second step of autophagy, phagophore nucleation (Hurley and Young, 2017). Western blot and DIA-MS analyses indicated a downregulation of total PIK3C3 protein, accompanied by an upregulation of phosphorylated PIK3C3 protein (Ser249), following GGH knockdown (Figure 3G; Supplementary Figure S3F). Upon GGH knockdown, the alteration of PIK3C3 mRNA level was not consistent with that of PIK3C3 protein level (Figure 3H; Supplementary Figure S3G). In addition, ATG14 and BECN1 protein levels increased, while their mRNA levels remained unchanged (Figure 3G, I, and J; Supplementary Figure S3H and I). The PIK3R4 protein level remained unaltered, while its RNA level decreased (Figure 3K; Supplementary Figure S3F and J). Thus, these results suggest that the activation of PI3KC3-C1 is independent of transcription regulation but rather depends on PIK3C3 phosphorylation and the increased ATG14 and BECN1 protein levels following GGH knockdown. Beyond lung cancer, knockdown of GGH could result in the activation of the ULK1 complex and PI3KC3-C1 via the phosphorylation of ULK1 and PIK3C3 in a multitude of cancer cell lines (Figure 3L).

During the late stage of autophagy, STX17 is one of the markers to drive the fusion of autophagosomes with late lysosomes (Tian et al., 2021). Our results indicated that neither the total or phosphorylated (Ser202) STX17 protein levels nor its RNA levels altered upon siGGH treatment (Figure 3M and N; Supplementary Figure S3K and L). These results suggest that GGH silencing could activate the ULK1 complex and PI3KC3-C1 to promote early autophagy rather than late autophagy.

### GGH knockdown promotes AMPKα activation via p-LKB1, p-CAMKK2, and AMP/ATP conversion

Next, we examined the expression and activation levels of several key regulators of autophagy upon GGH knockdown.

**Figure 4:**
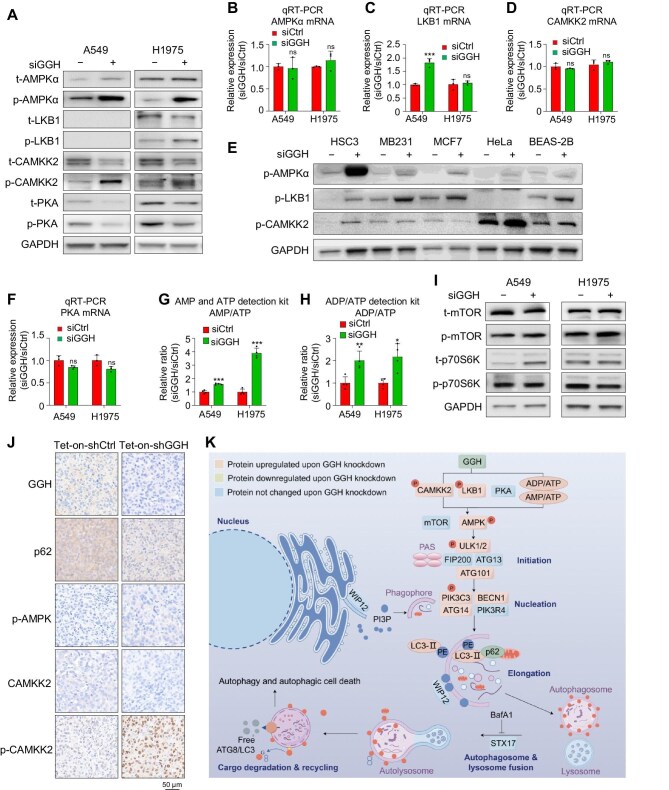
GGH knockdown activates AMPK, CAMKK2, and LKB1. (**A**) Western blot analysis of t-AMPKα, p-AMPKα (THR172), t-LKB1, p-LKB1 (Ser428), t-CAMKK2, p-CAMKK2 (Ser511), t-PKA C-α, and p-PKA C-α (Thr197) after 72 h siGGH treatment. (**B**–**D**) AMPKα, LKB1, and CAMKK2 mRNA expression after siGGH treatment. Data are shown as mean ± SD, *n* = 3. ***P < 0.001. (**E**) Western blot analysis of p-AMPKα (THR172), p-LKB1 (Ser428), and p-CAMKK2 (Ser511) proteins after 72 h siCtrl or siGGH treatment in diverse cancer cell lines and NBE cell line. (**F**) PKA mRNA expression after siGGH treatment. Data are shown as mean ± SD, *n* = 3. (**G** and **H**) AMP/ATP and ADP/ATP ratios after siCtrl or siGGH transfection in A549 and H1975 cell lines. Data are shown as mean ± SD, *n* = 4. *P < 0.05, **P < 0.01, ***P < 0.001. (**I**) Western blot analysis of t-mTOR, p-mTOR (Ser2448), t-p70S6K, and p-p70S6K (Thr389) after 72 h siGGH treatment. (**J**) IHC staining of mouse tumor sections showing the expression of GGH, p62, p-AMPK (Thr172), CAMKK2, and p-CAMKK2 (Ser511). Scale bar, 50 μm. (**K**) A schematic illustrating how silencing GGH promotes early autophagy (created by Figdraw).

AMPKα has been reported to trigger autophagy by activating ULK1 at Ser555 (Lindqvist et al., 2018). We observed a weak elevation in total AMPKα protein expression but more pronounced elevation in phosphorylated AMPK protein (Thr172) expression after siGGH treatment (Figure 4A; Supplementary Figure S4A), without significant changes in the AMPKα mRNA level (Figure 4B; Supplementary Figure S4B), suggesting that AMPK activation may play a significant role in siGGH-induced autophagy.

We further investigated three major kinases responsible for AMPK activation, serine/threonine kinase 11 (STK11 or LKB1), calcium/calmodulin-dependent protein kinase kinase 2, beta (CAMKK2 or CaMKKβ), and protein kinase A (PKA) (Long and Zierath, 2006; Huang et al., 2019). Previous studies indicated that p-LKB1 and p-CAMKK2 are p-AMPK inducers, while PKA serves as an inhibitor (Grisan et al., 2021; Wani et al., 2021). In our study, western blot analysis revealed the downregulation of total LKB1 or CAMKK2 protein and upregulation of p-LKB1 (Ser428) or p-CAMKK2 (Ser511) protein following GGH knockdown in A549 (LKB1 mutant) and H1975 cell lines (Figure 4A; Supplementary Figure S4A). These changes in LKB1 and CAMKK2 protein levels did not correspond to the alterations in their mRNA levels (Figure 4C and D; Supplementary Figure S4C and D), indicating that the activation of LKB1 and CAMKK2 mainly involves post-translational modifications, especially phosphorylation. GGH silencing could also activate AMPK through LKB1 and CAMKK2 phosphorylation in various cancer cell lines and normal lung cells (Figure 4E). Meanwhile, we observed that siGGH treatment reduced the expression of both total and phosphorylated PKA (Thr197) proteins but did not change the PKA mRNA level (Figure 4A and F; Supplementary Figure S4A and E), indicating that PKA may be involved in GGH-related autophagy.

Nutrient or energy starvation induces autophagy, which depends heavily on the precise regulation of ATP synthesis and degradation (ATP ↔ ADP + phosphate, ADP → AMP + phosphate) (Bullon et al., 2016). AMPK acts as an energy sensor, responding to changes in the cellular levels of ATP, ADP, and AMP. When cellular energy levels are low, indicated by a high AMP/ATP or ADP/ATP ratio, AMPK is activated (phosphorylated) to restore energy balance by promoting catabolic pathways that generate ATP and inhibiting anabolic pathways that consume ATP (Hardie et al., 2012; Park et al., 2023). Here, both AMP/ATP and ADP/ATP ratios were significantly increased upon siGGH treatment in A549 and H1975 cells (Figure 4G and H; Supplementary Figure S4F).

The mTOR complex 1, a key negative regulator of autophagy, inhibits the function of AMPKα by disrupting the interaction between AMPKα and ULK1 (Park et al., 2023). However, the protein and mRNA levels of mTOR and its downstream target p70S6K were not changed by siGGH treatment (Figure 4I; Supplementary Figure S4G−K). These results indicate that GGH silencing might induce autophagy through increasing the ratio of ADP/ATP or AMP/ATP and activating LKB1 and CAMKK2, which lead to the activation of AMPKα but not mTOR.

To determine the effects of targeting GGH in a mouse subcutaneous tumor model, nude mice were subcutaneously injected with the stable A549 cell line where doxycycline (Dox) induces the expression of Tet-on-shCtrl or Tet-on-shGGH (Supplementary Figure S4L) and fed a Dox-containing diet (200 mg/kg). Mice with Tet-on-shGGH cells exhibited a marked reduction in tumor growth compared to those with Tet-on-shCtrl cells (Supplementary Figure S4M). Subsequent immunohistochemistry (IHC) staining of the xenografted tumor tissues demonstrated that shGGH effectively knocked down GGH expression, suppressed p62 level, and promoted p-AMPK and p-CAMKK2 levels (Figure 4J). Thus, GGH knockdown could also trigger autophagy of tumor cells *in vivo*.

In summary, silencing GGH might activate AMPKα by raising the AMP/ATP or ADP/ATP ratio, activating LKB1 and CAMKK2, and inhibiting PKA, which subsequently triggers early autophagy through the ULK1 complex and PI3KC3-C1, ultimately resulting in autophagic cell death (Figure 4K).

### GGH affects autophagy through NADH

**Figure 5:**
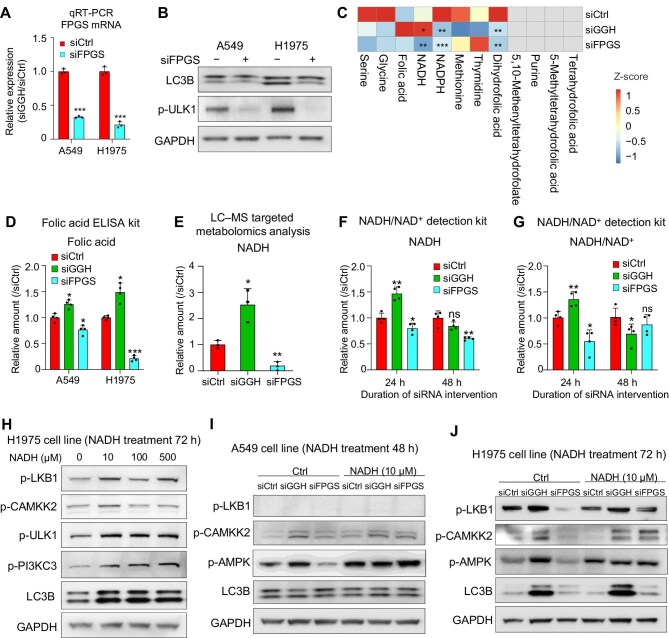
Silencing GGH promotes autophagy through NADH. (**A**) FPGS mRNA expression was significantly reduced after siFPGS treatment in A549 and H1975 cell lines. (**B**) Western blot analysis of LC3B and p-ULK1 (Ser555) after 72 h siFPGS treatment. (**C**) Levels of potential metabolites involved in folate metabolism measured by LC–MS targeted metabolomics in A549 cell line. Data were obtained from three repeated measurements. (**D**) Relative folic acid amount after siCtrl, siGGH, or siFPGS transfection in A549 and H1975 cell lines measured by the folic acid ELISA kit. Data are shown as mean ± SD, *n* = 4, *P < 0.05, ***P < 0.001. (**E**) LC–MS targeted metabolomics analysis of relative NADH amount after siCtrl, siGGH, or siFPGS transfection in A549 cell line. Data are shown as mean ± SD, *n* = 3, *P < 0.05, **P < 0.01. (**F** and **G**) Relative NADH amount and NADH/NAD^+^ ratio after siCtrl, siGGH, or siFPGS transfection in A549 cell line measured by the NADH/NAD^+^ detection kit. Data are shown as mean ± SD, *n* = 4, *P < 0.05, **P < 0.01. (**H**) Western blot analysis showing changes in autophagy-related protein expression after 0, 10, 100, or 500 μM NADH treatment in H1975 cell line. (**I** and **J**) Western blot analysis showing changes of autophagy-related protein expression after siRNA and/or 10 μM NADH treatment in A549 and H1975 cell lines.

Folylpolyglutamate synthetase (FPGS) plays an essential role in folate polyglutamation and intracellular folate accumulation, which is opposite to the role of GGH in folate metabolism (Zheng and Cantley, 2019). Indeed, in contrast to promoting autography by silencing GGH, silencing FPGS inhibited LC3B-I/II conversion and ULK1 activation (Figure 5A and B). Therefore, GGH may work as a folate metabolism-related enzyme to affect autography.

Liquid chromatography–mass spectrometry (LC–MS) targeted metabolomics analysis revealed that the folate (also named folic acid) level in A549 cells increased upon GGH knockdown but decreased upon FPGS knockdown, although not statistically significant probably due to the large variability among the data points (Figure 5C). Using a folic acid enzyme-linked immunosorbent assay (ELISA) kit, we confirmed that knocking down GGH significantly increased folate levels, while knocking down FPGS significantly decreased folate levels (Figure 5D). However, knocking down GGH or FPGS did not affect glutamate levels in cells (Supplementary Figure S5A). These results indicate that GGH knockdown may promote autophagy through increasing polyglutamated folate stress.

LC–MS targeted metabolomics analysis also showed that NADH, NADPH, and dihydrofolic acid levels were significantly altered upon siGGH or siFPGS treatment in A549 cells (Figure 5C). In particular, NADH amount in the siGGH group was significantly higher than that in the siCtrl group, while the amount in the siFPGS group was significantly lower (Figure 5E). Using an NADH/NAD^+ ^detection kit, we validated the increased NADH amount and NADH/NAD^+^ ratio in the siGGH group and the decreased NADH amount and NADH/NAD^+^ ratio in the siFPGS group after 24 h of siRNA transfection (Figure 5F and G). Based on these results, we speculated that GGH and FPGS could regulate polyglutamated folate stress within cells and adjust NADH levels, thereby modulating autophagy.

Then, we treated A549 and H1975 cells with various concentrations of NADH. Western blot analysis demonstrated that 72 h NADH treatment increased p-LKB1, p-CAMKK2, p-AMPK, p-ULK1, and p-PI3KC3 levels, elevated LC3B-II/I conversion, and decreased p62 level (Figure 5H; Supplementary Figure S5B). Moreover, 10 μM NADH treatment already caused a notable decrease in cell number accompanied by deteriorated cellular status, resembling the phenotype upon GGH knockdown (Supplementary Figures S5C and S1), indicating that NADH could induce early autophagy. Furthermore, NADH administration rescued the decreased p-CAMKK2, p-LKB1, and p-AMPK levels and LC3B-I/II conversion by FPGS knockdown, which was similar to the effect following GGH knockdown, in A549 (LKB1 mutant) and H1975 cell lines (Figure 5I and J). In conclusion, knocking down GGH can elevate polyglutamated folate stress and NADH levels within cells, thereby modulating autophagy, while knocking down FPGS has the opposite effect. When the reduced levels of NADH following FPGS knockdown are replenished, autophagy is reinstated, mirroring the phenomenon observed upon GGH knockdown.

## Discussion

In this study, we offered new insights into the function, molecular mechanisms, and clinical significance of GGH. We demonstrated that reduced GGH expression could induce autophagy and autophagic cell death in a wide variety of cancer and lung epithelial cells. Furthermore, we found that silencing GGH could activate the ULK1 complex and PI3KC3-C1 via AMPK, promoting early autophagy. The activation of AMPK is driven by multiple factors, including the activation of LKB1 and CAMKK2 and the increased AMP/ATP and ADP/ATP ratios. Additionally, the folate synthetase FPGS functions opposite to GGH in the regulation of folate metabolism and also autophagy, suggesting that folate stress plays an essential role in GGH knockdown-induced early autophagy. Among the potential metabolites involved in folate metabolism, we focused on NADH.

NAD is a ubiquitous cellular coenzyme located in the cytosol and mitochondria (Berger et al., 2004). NAD primarily functions as an electron acceptor by shuttling between its oxidized form (NAD^+^) and reduced form (NADH). It is known that NADH accumulation is toxic, causing blockages to TCA turning and impairment of *de novo* aspartate synthesis, resulting in decreased protein and nucleotide synthesis (Yang et al., 2020). Increasing the NADH/NAD^+^ ratio has been shown to decrease cell proliferation (Luengo et al., 2021). Moreover, recent studies suggested that NAD, particularly its reduced form (NADH), may play a regulatory role in autophagy. One study found that FK866 decreases total NAD levels and increases NADH/NAD^+^ ratios, which is associated with decreased oxygen consumption, decreased ATP production, and increased glycolytic gene expression in rat primary cardiomyocytes (Oyarzún et al., 2015). Another study found that FK866 inhibits total NAD by hindering nicotinamide phosphoribosyl transferase, leading multiple myeloma cells to undergo autophagy or autophagic cell death (Billington et al., 2008). The administration of NAD reduces ischemic brain damage and blocks post-ischemic autophagy in brain ischemia (Zheng et al., 2012). Fang et al. (2019) found that mitophagy is blocked in Werner syndrome and restored by NAD(H) replenishment through AD^+^ precursor nicotinamide riboside in a DCT-1 (NIX) and UNC-51 (ULK1)-dependent manner. These findings support the notion that NADH plays a major role in inducing autophagy. Our results align with these studies, indicating that NADH induces autophagy.

Folate acts as a source of NADH. Generally, NAD is synthesized via two pathways: the *de novo* pathway (from tryptophan) and the salvage pathway (Houtkooper et al., 2010). Yang et al. (2020) reported that folate-dependent serine catabolism through MTHFD2 also generates abundant NADH. They demonstrated that as the NADH/NAD^+^ ratio increases, other NADH sources are suppressed, thereby establishing folate-dependent serine catabolism as the primary source (Yang et al., 2020). In this study, we focused on the folate hydrolase GGH, which hydrolyzes all forms of folate, facilitating their excretion from cells and reducing intracellular folate pressure. Consequently, GGH deficiency elevates NADH levels by increasing folate pressure. However, there are only a few reports on the role of folate in autophagy. Folate has been found to induce autophagy through the mTOR–SREBP-1–PI3K pathway and AMPK/mTOR signaling (Onodera et al., 2014; Zhang et al., 2021). Our findings provide a novel and insightful perspective on the mechanism of folate-induced autophagy mediated by NADH.

The potential mechanisms by which NADH induces autophagy include the following ones. (i) During the TCA cycle, NADH is converted into NAD^+^ at mitochondrial respiratory complex I. In stressed states, elevated mitochondrial NADH/NAD^+^ ratios lead to ROS production and mitochondrial dysfunction. KGDH and PDH are also mitochondrial ROS producers that depend on NADH. Excessive amounts of ROS have been shown to cause oxidative injury and autophagy (Xiao et al., 2018). (ii) NAD^+^ is a key regulator of ATP generation. In the cytosol, NAD^+^/NADH redox pairs support ATP production through glycolysis (Bogorad et al., 2013). In the mitochondria, the NAD^+^/NADH redox pair contributes to ATP production by modulating acetyl CoA generation, TCA cycle processes, and NADH oxidation via the mitochondrial respiratory complex I (Zhang et al., 2016). Therefore, NADH may induce autophagy by causing ATP depletion and energy starvation. (iii) The NAD^+^/NADH balance directly influences intracellular calcium regulation, which is correlated with autophagy, e.g. NADH interacts with dopamine to increase Ca^2+^ signals (Wang et al., 2012). (iv) The expression levels of three main types of proteins consume NAD, i.e. PARPs, sirtuins, and NAD glycohydrolases (CD38 and CD157) (Xiao et al., 2018), and NAD(H) levels are mutually regulated. For instance, an increased NADH/NAD^+^ ratio activates sirtuin expression, while cellular NADH/NAD^+^ levels are also regulated by these deacetylases (SIRT1–7) (Gambini et al., 2011). PARPs, sirtuins, and CD38 are well-known autophagy inducers, capable of triggering autophagy through various pathways at different stages (Kim et al., 2022; Wilson et al., 2023). Thus, a high NADH/NAD^+^ ratio might induce autophagy via these NAD-consuming enzymes.

In summary, this study sheds light on how GGH deficiency elevates NADH levels and induces early autophagy and autophagic cell death. It also establishes the association between folate stress and NADH and proposes a therapeutic strategy for LUAD through autophagy induction by targeting GGH or NADH intervention. Further studies are needed to determine how folate stress affects the NADH/NAD^+^ ratio and how exactly NADH influences autophagy. Notably, after siGGH transfection, ULK1 is not merely activated by AMPK but also shows a marked upregulation of its mRNA expression. Future research should investigate how GGH regulates ULK1 at the transcriptional level. The modulation of the NADH/NAD^+^ ratio represents a dynamic process, and thus further exploration is required to understand how it regulates autophagy in tumor cells, as well as the optimal timing and dosage of NADH intervention. Moreover, the role of GGH in tumors, besides regulating autophagy, needs further exploration. Answers to these questions will help understand the roles of folate metabolic pathways and NADH/NAD^+^ energy homeostasis in cancers and better design tailored therapeutic strategies.

## Materials and methods

### Cell lines

Human non-small cell lung cancer cell lines A549, H1975, and H838 and TNBC cell line MDA-MB-231 were purchased from Cell Bank of the Chinese Academy of Sciences. Lung epithelial cell line BEAS-2B and human OSCC cell line HSC3 were kindly gifted by Prof. Jian Zhang (Southern University of Science and Technology). CC cell line HeLa was kindly gifted by Prof. Jintang Dong (Southern University of Science and Technology). ER^+^ BC cell line MCF7 was kindly gifted by Prof. Xin Hong (Southern University of Science and Technology). All cell lines are confirmed by short-tandem repeat analysis.

### siRNA transfection

A549 and H1975 cells were seeded overnight in 6-well plates. For transfection, siCtrl, siGGH, or siFPGS and Lipofectamine RNAiMAX Reagent (Invitrogen) were separately incubated with 100 μl of siRNA transfection reagent and incubated for 5 min at room temperature, then combined and incubated for an additional 15 min, and added to the cells. After 48  or 72 h, cells were collected for further experiments. The siRNA reagents are listed in Supplementary Table S1.

### Western blot analysis

After protein quantification, denatured proteins were loaded and separated on 4%–20% sodium dodecyl sulphate–polyacrylamide gel electrophoresis gradient gels. The proteins were transferred to the polyvinylidene difluoride membrane at 200 mA for 2 h and then blocked with 1% bovine serum albumin for 1 h. Primary antibody incubation was performed overnight at 4°C, followed by incubation with secondary antibodies for 1 h at room temperature. Except for the GGH antibody (ABclonal Technology Co.), all other antibodies were purchased from Cell Signaling Technology. Finally, enhanced chemiluminescence solution was added, and images were captured using a western blot fluorescence imager. The antibodies are listed in Supplementary Table S1.

### Colony formation assay

A549 and H1975 cells were seeded overnight in 24-well plates. After 24 h of siRNA transfection, cells were digested and counted using a cell counting plate. Subsequently, 300–800 cells were reseeded in 6-well plates and cultured for 10–12 days. siRNAs were refreshed during the incubation period.

### RFP-GFP-LC3B assay

The RFP-GFP-LC3 assay was used to monitor autophagic flux. After 24 h of siRNA transfection, mRFP-GFP-LC3 tandem fluorescent protein adenovirus was added to each well with half-volume 1640 complete medium. After 4 h, the medium was supplemented to its original volume. Eight hours after infection, the virus-containing medium was replaced with fresh 1640 complete medium for continued culture. Fluorescence images were captured 48 h post-virus incubation using a Leica SP8 microscope. Puncta of autophagosomes and autolysosomes were counted in five images per well at 400× magnification.

### DIA-MS

After protein extraction and preparation, LC separation was performed using an UltiMate 3000 LC system (Thermo Fisher Scientific). DIA data were acquired in the diaPASEF mode. The DIA data were processed and analyzed using Spectronaut 18 (Biognosys AG) with default settings.

### RNA-seq

After 48 h of siRNA transfection, RNA was extracted using a TRIzol Reagent kit (Invitrogen). After total RNA extraction, mRNA was enriched using Oligo (dT) beads and reverse-transcribed into cDNA. The cDNA fragments were then purified with a QiaQuick PCR extraction kit (Qiagen), end-repaired, poly(A)-added, and ligated to Illumina sequencing adapters. Sequencing was performed on an Illumina HiSeq2500 by Guangzhou Gene Denovo Biotechnology Co., Ltd. Quality control and preprocessing were applied to the raw sequencing data. Fragments per kilobase million (FPKM) were calculated to quantify transcription expression abundance and variation using StringTie software.

### qRT-PCR

Total RNA was extracted according to the kit's instructions (Vazyme). After quantification using NanoDrop 2000, total RNA was reverse-transcribed using the HiScript II Q RT SuperMix for qPCR Kit (Vazyme) according to the instructions. The resulting product was analyzed by qRT-PCR using Taq Pro Universal SYBR qPCR Master Mix (Vazyme). Primers are listed in Supplementary Table S1.

### LC–MS targeted metabolomics analysis

Targeted metabolomics analysis was conducted by Guangzhou Gene Denovo Biotechnology Co., Ltd. After sample preparation, LC–MS experiments were performed using Waters ACQUITY UPLC and AB SCIEX 5500 QQQ-MS with PREMIER BEH Z-HILIC. Data were processed using Multiquant software.

### ATP/ADP detection

A549 and H1975 cells were seeded overnight in 96-well plates. After 72 h of siRNA transfection, ADP/ATP ratio in each well was assessed using the ADP/ATP Ratio Assay Kit (Sigma). Fluorescent signals were detected using a fluorescence microplate reader (BioTek Synergy HTX). Each group contained six replicates.

### NADH/NAD^+^ detection

A549 and H1975 cells were seeded overnight in 96-well plates. After 24 or 48 h of siRNA transfection, NADH and total NAD levels in each well were assessed using an NAD^+^/NADH detection kit (Beyotime) and determined spectrophotometrically at 340 nm (BioTek Synergy HTX). Each group contained six replicates.

### ATP content assay

A549 and H1975 cells were seeded overnight in 6-well plates. After 72 h of siRNA transfection, ATP content in each well was assessed using an ATP Content Assay kit (Solarbio) and determined spectrophotometrically at 340 nm (BioTek Synergy HTX). Each group contained four replicates.

### AMP-Glo^TM^ assay

A549 and H1975 cells were seeded overnight in 6-well plates. After 72 h of siRNA transfection, AMP content in each well was assessed using an AMP Content Assay kit (Promega). The luminescence activity was quantified using a Promega GloMax Navigator microplate luminometer. Each group contained four replicates.

### Glutamic acid content assay

A549 and H1975 cells were seeded overnight in 6-well plates. After 72 h of siRNA transfection, glutamic acid content in each well was assessed using the Glutamic Acid (Glu) Content Assay Kit (Solarbio) and determined spectrophotometrically at 340 nm (BioTek Synergy HTX). Each group contained four replicates.

### Folic acid/vitamin B9 ELISA

A549 and H1975 cells were seeded overnight in 6-well plates. After 48 h of siRNA transfection, cells were gently washed with cold phosphate-buffered saline (PBS) and subsequently digested with trypsin. After centrifugation at 1000× *g* for 5 min, the cells were collected, resuspended in PBS (containing a protease inhibitor at a ratio of 100:1), and lysed using ultrasound. After centrifugation at 1500× *g* for 10 min at 4°C, the supernatant was collected for detection. Folic acid concentration in each well was assessed using the FA/VB9 (Folic Acid/Vitamin B9) ELISA Kit (Elabscience) and determined spectrophotometrically at 450 nm (BioTek Synergy HTX). Each group contained four replicates.

### Animal experiments

The stable A549 cell lines were generated through infecting with TetIIP-Turbo RFP-MCS (MIR30)-Ubi-TetR-IRES-Puromycin lentiviruses. Cells were subcutaneously injected into 5-week-old BALB/c nude mice, and the mice were fed a diet containing Dox at a concentration of 200 mg/kg, supplied by Jiangsu Xietong Pharmaceutical Bio-engineering Co., Ltd. Tumor sizes were monitored every other day over a total period of 20 days. The mouse model utilized in this study was derived from our previously unpublished research.

### Statistical analysis

Data were graphed using GraphPad Prism and Microsoft Excel software. Survival curves were estimated using Kaplan–Meier analysis and compared using the log-rank test. Differential gene expression of RNA-seq was determined using DESeq2. Statistical significance was calculated using an unpaired two-tailed Student's *t*-test, with a *P*-value <0.05 considered statistically significant.

### Data availability

GDC TCGA LUAD and TCGA TARGET GTEx data were downloaded from UCSC Xena (https://xenabrowser.net/). The Gillette dataset is available at the CPTAC Data Portal (https://cptac-data-portal.georgetown.edu/cptacPublic/).

## Supplementary Material

mjaf014_Supplemental_File

## Notes

**Author contributions:** Y.L. and G.C. conceived and designed the experiments and wrote the original draft; Y.L., Y.D., S.C., Z.X., X.L., B.L., Z.Z., and H.Z. performed the experiments and analyzed data; and G.C. was in charge of conceptualization, supervision, and funding acquisition. All authors read and approved the final manuscript.
