# Supplementary material for: Silencing GGH induces autophagy by increasing folate stress and production of NADH
Source: J Mol Cell Biol. 2025 Apr 23;17(3):mjaf014. doi: 10.1093/jmcb/mjaf014 (PMC12527274; doi:10.1093/jmcb/mjaf014)
Supplement: mjaf014_Supplemental_File [file mjaf014_supplemental_file.pdf]

Silencing GGH induces autophagy by increasing folate stress and production of NADH

Yu Li[1], Yuhui Du[1], Sijie Chen[1], Zhangrong Xie[1], Xinrui Li[1], Baoyue Lin[1,2], Zhiqing Zhou[1], Huijie Zhao[1,3], Guoan Chen[1,4,5]

[1]Department of Human Cell Biology and Genetics, Joint Laboratory of Guangdong-Hong Kong Universities for Vascular Homeostasis and Diseases, School of Medicine, Southern University of Science and Technology, Shenzhen, Guangdong 518055, China;

[2]Department of Critical Care Medicine, The First Affiliated Hospital, Zhejiang University School of Medicine, Hangzhou 311399, China;

[3]Department of Oncology, Sun Yat-sen Memorial Hospital, Sun Yat-sen University, Guangzhou 510120, China;

[4]The First Affiliated Hospital of Southern University of Science and Technology, Shenzhen 518055, China;

[5]SUSTech Homeostatic Medicine Institute, School of Medicine, Southern University of Science and Technology, Shenzhen 518055, China

Corresponding authors: Guoan Chen, Department of Human Cell Biology and Genetics, Joint Laboratory of Guangdong-Hong Kong Universities for Vascular Homeostasis and Diseases, School of Medicine, Southern University of Science and Technology, Shenzhen, Guangdong 518055, China, E-mail: cheng@sustech.edu.cn

Supplementary Figure S1

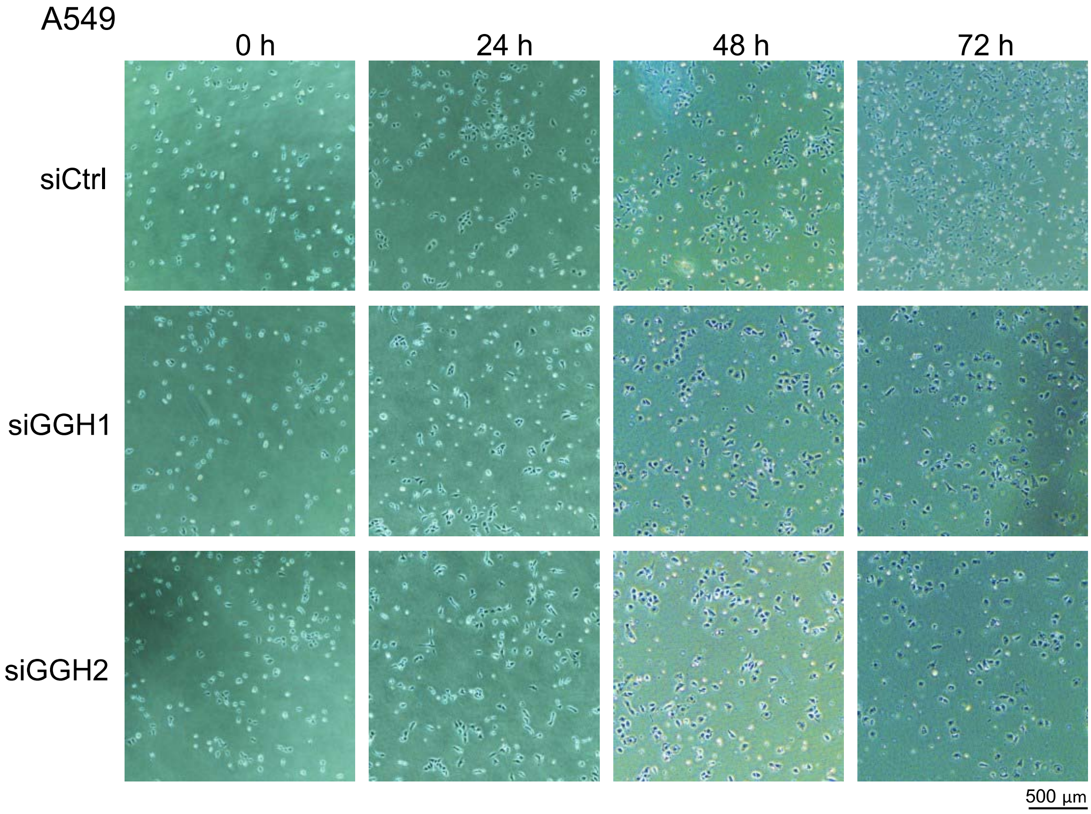

Figure S1. Representative photograph (4x magnification, scale bar represented 500 µm) showing changes in cell number at 24 hours, 48 hours, and 72 hours following GGH siRNAs transfection in A549 cell line.

Supplementary Figure S2

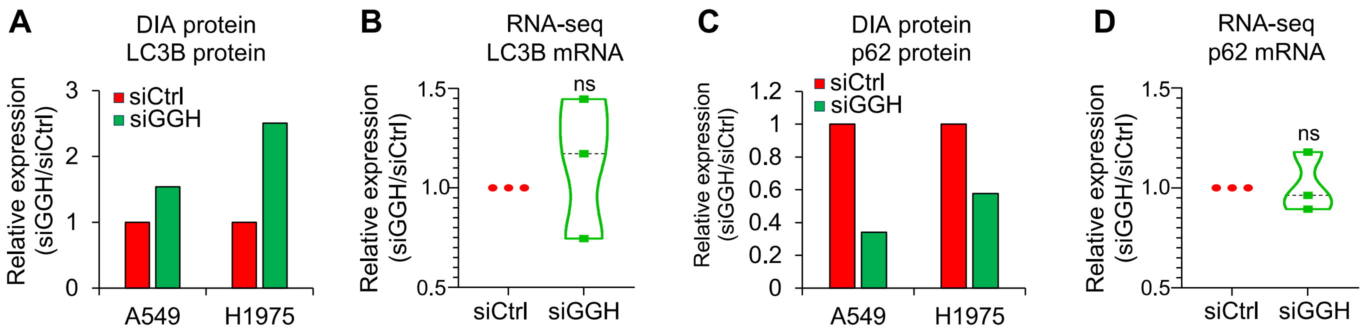

Figure S2. Changes in LC3B and p62 proteins and RNA expression levels upon siGGH transfection. (**A**) DIA-MS showing LC3B protein levels. (**B**) RNA-seq showing LC3B RNA levels in A549, H1975 and H838 cell lines. (**C**) DIA-MS showing p62 levels. (**D**) RNA-seq showing p62 RNA levels in A549, H1975 and H838 cell lines. Differential gene expression of RNA-seq was determined using DESeq2.

Supplementary Figure S3

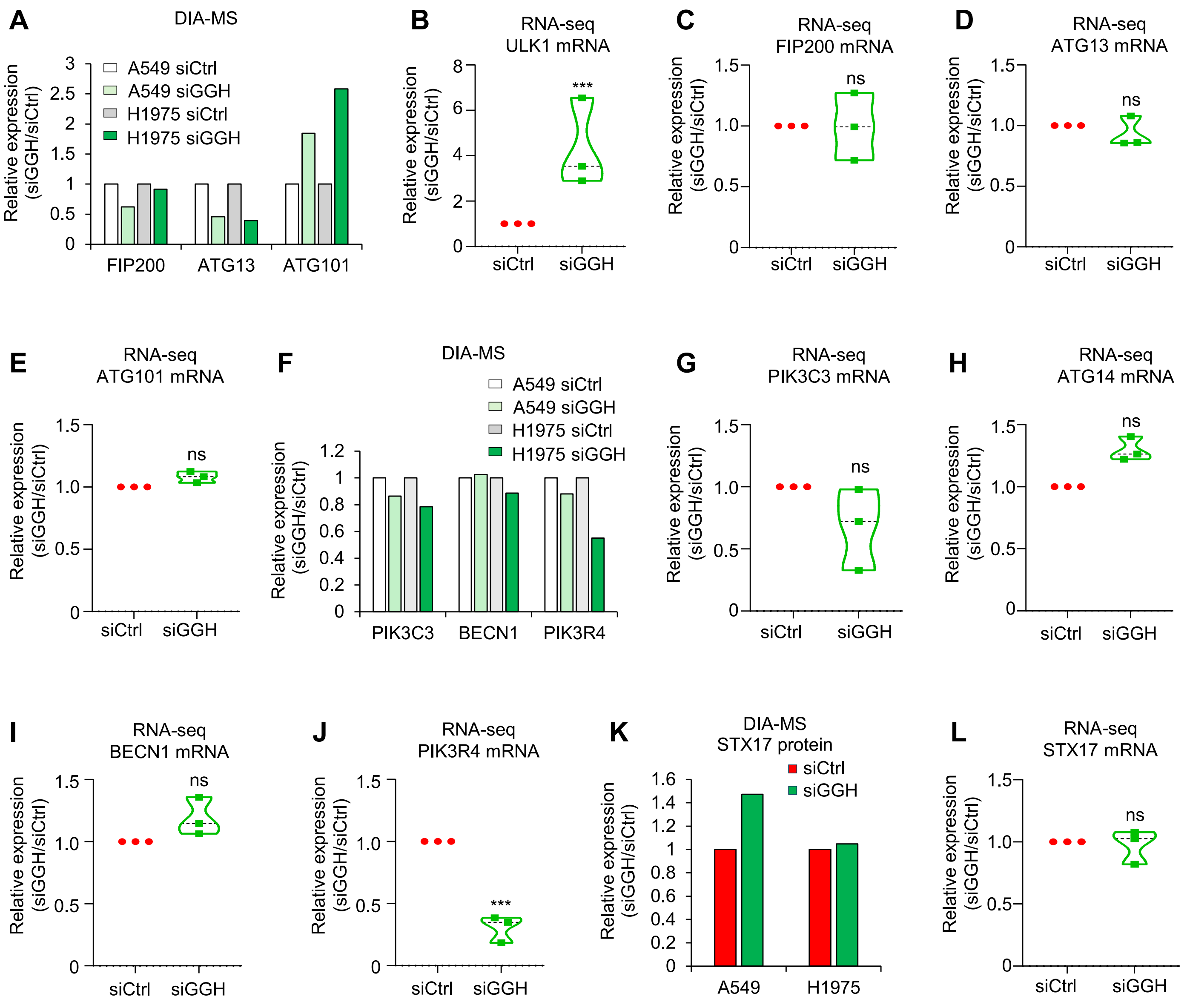

Figure S3. Changes in ULK1/ATG1 complex, PI3KC3-C1 and STX17 protein and RNA expression levels upon siGGH transfection. (**A**) DIA-MS showing protein expression levels of ULK1/ATG1 complex. (**B-E**) RNA-seq showing RNA expression levels of ULK1/ATG1 complex in A549, H1975 and H838 cell lines. *** *P* < 0.001. (**F**) DIA-MS showing protein expression levels of PI3KC3 complex 1. (**G-J**) RNA-seq showing RNA expression levels of PI3KC3 complex 1 in A549, H1975 and H838 cell lines. *** *P* < 0.001. (**K, L**) DIA-MS and RNA-seq showing protein and RNA expression levels of STX17. Differential gene expression of RNA-seq was determined using DESeq2.

Supplementary Figure S4

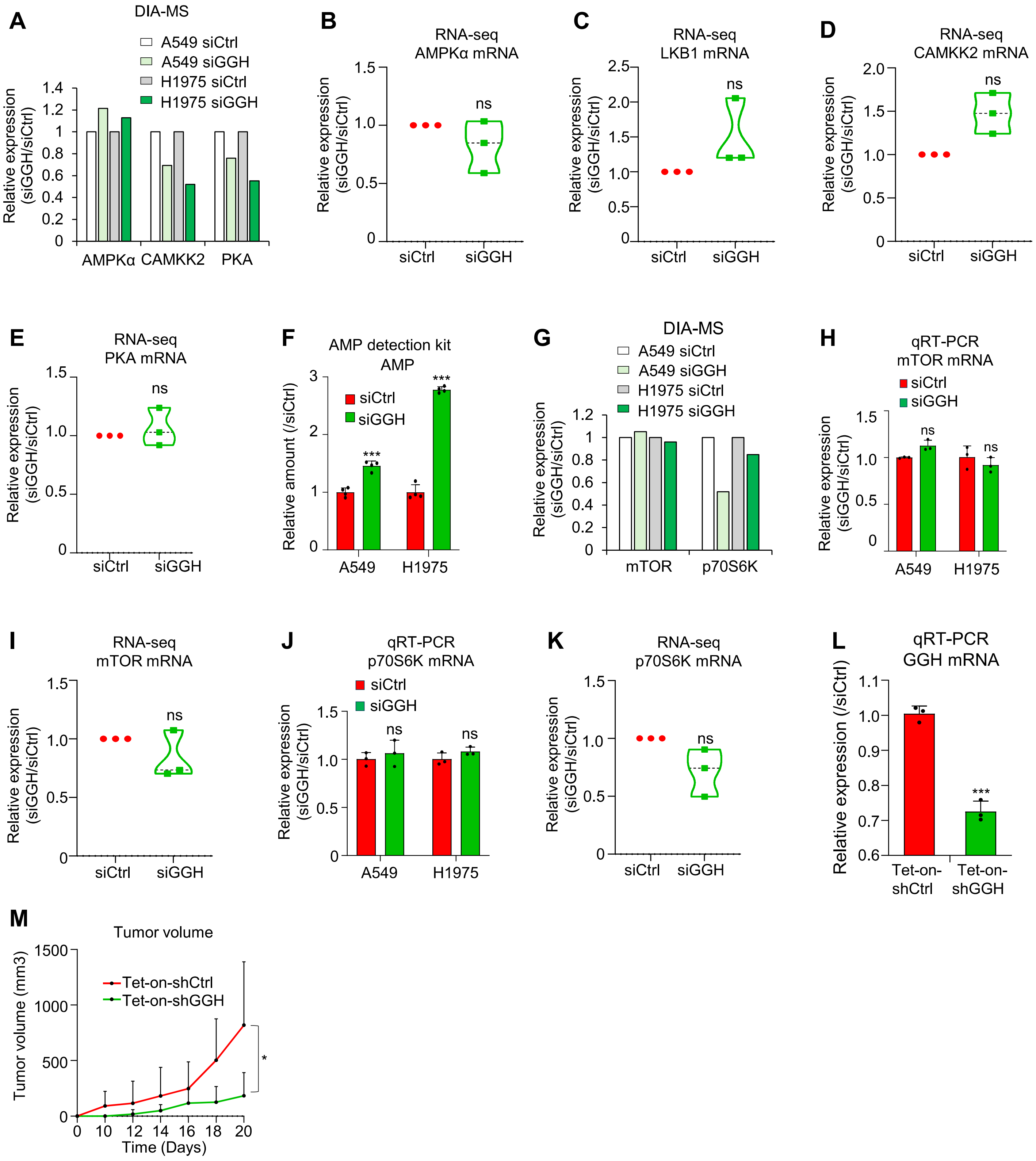

Figure S4. Changes in AMPK, CAMKK2, LKB1, PKA, mTOR and p70S6K protein and RNA expression levels upon siGGH transfection. (**A**) DIA-MS showing protein expression levels of AMPK, CAMKK2 and PKA. (**B-E**) RNA-seq showing RNA expression levels AMPK, CAMKK2, LKB1 and PKA in A549, H1975 and H838 cell lines. (**F**) The relative amount of AMP after siCtrl and siGGH transfection in A549 and H1975 cell lines. Data were shown as mean ± SD, n = 4, *** $P < 0.001$. (**G-K**) DIA-MS, qRT-PCR and RNA-seq showing protein and RNA expression levels of mTOR and P70S6K. Differential gene expression of digital RNA-seq was determined using DESeq2. Data were shown as mean ± SD, n = 3. (**L**) Tet-on-shGGH knockdown efficiency was measured by qRT-PCR. Tetracycline (Tet)-inducible stable A549 cell lines express shRNAs under 10 μg/ml doxycycline (Dox), either non-targeting (Tet-on-shCtrl) or targeting GGH (Tet-on-shGGH). Data were shown as mean ± SD, n = 3, *** $P < 0.001$. (**M**) The volume of the tumor-bearing tissues was systematically monitored and calculated. Following tumor inoculation in nude mice, the dimensions of the tumors were measured bi-daily over a total observation period of 20 days. The mouse model utilized in this study was derived from our previously unpublished research (Yu Li, 2025, Unpublished Data). Data were shown as mean ± SD, n = 5, * $P < 0.05$.

Supplementary Figure S5

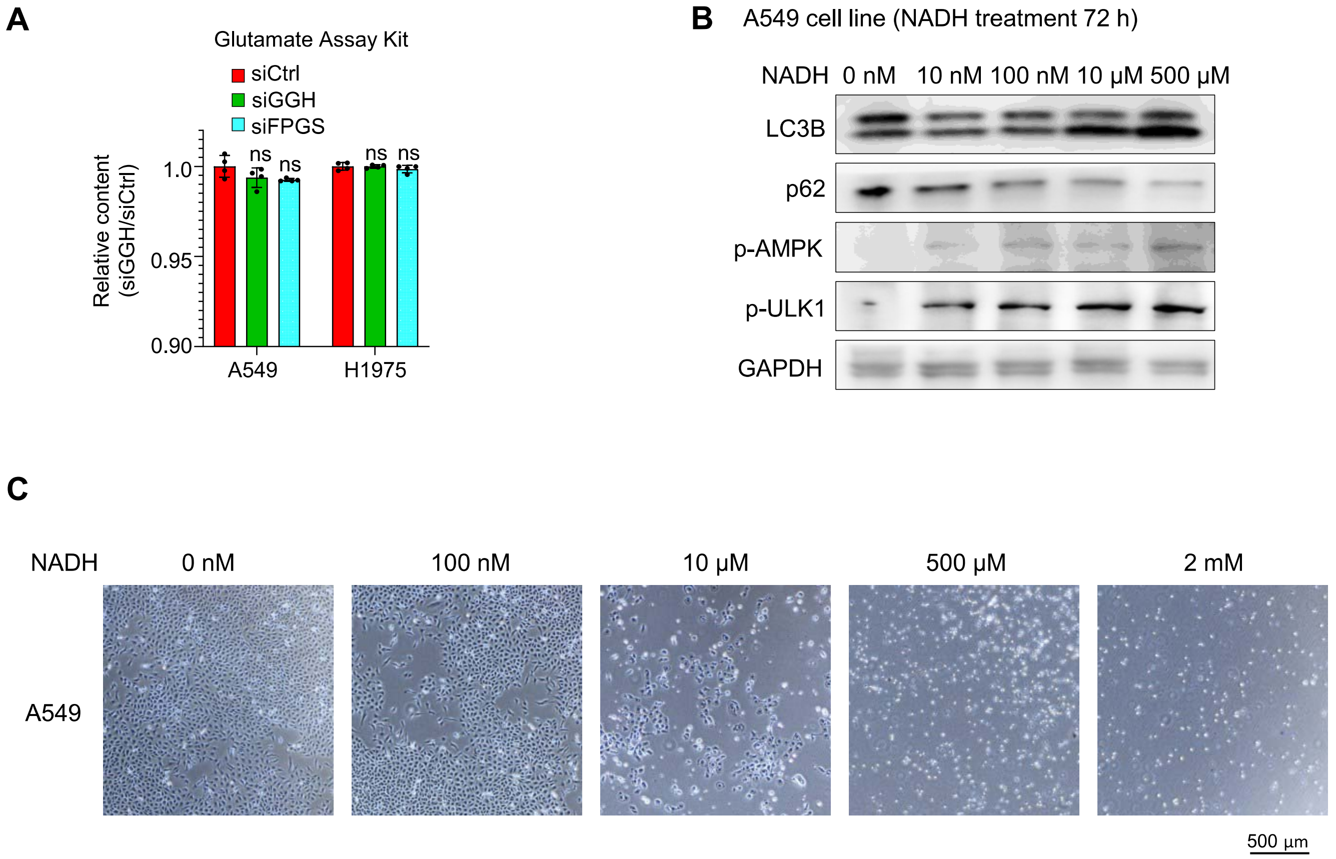

Figure S5. Silencing of GGH does not affect glutamate and NADH could induce early autophagy. (**A**) Relative Glutamate levels are measured after siCtrl, siGGH and siFPGS transfection at 72 h in A549 cell line using the Glutamate Assay Kit. Data were obtained from 4 repeated measurements and shown as mean ± SD. (**B**) Western blot showing the changes in GGH and autophagy-related protein expression treated with different concentrations of NADH in A549. (**C**) Representative photograph (4x magnification, scale bar represented 500 μm) showing cellular state change treated 0 nM, 100 nM, 10 μM, 500 μM and 2 mM NADH in A549.

Supplementary Table S1. siRNA sequence，PCR primer and regents

Sequence (5'-3')	Source	siRNA
UUCUCCGAACGUGUCACGUTT	GenePharma	Ctrl siRNA target sequence-sense
ACGUGACACGUUCGGAGAATT	GenePharma	Ctrl siRNA target sequence-antisense
CCUCAGACGCUCAGAUUAUTT	GenePharma	GGH1 siRNA target sequence-2-sense
AUAAUCUGAGCGUCUGAGGTT	GenePharma	GGH1 siRNA target sequence-2-antisense
CCAGUGGCAUCCAGAGAAATT	GenePharma	GGH2 siRNA target sequence-2-sense
UUUCUCUGGAUGCCACUGGTT	GenePharma	GGH2 siRNA target sequence-2-antisense
GGGCUUAUGACUGCACCAATT	GenePharma	FPGS1 siRNA target sequence-1-sense
UUGGUGCAGUCAUAAGCCCTT	GenePharma	FPGS1 siRNA target sequence-1-antisense
GGGAUACGGUGGAGAAGAUTT	GenePharma	FPGS2 siRNA target sequence-2-sense
AUCUUCUCCACCGUAUCCCTT	GenePharma	FPGS2 siRNA target sequence-2-antisense
GCAGAUCUCAUGUCCUCUATT	GenePharma	FPGS3 siRNA target sequence-2-sense
UAGAGGACAUGAGAUCUGCTT	GenePharma	FPGS3 siRNA target sequence-2-antisense
**Sequence**	**Source**	**RT-PCR primers**
GGCTGTTGTCATACTTCTCATGG	BGI Genomics	GAPDH F
GGAGCGAGATCCCTCCAAAAT	BGI Genomics	GAPDH R
TCAACAATGGAAGTTTTCCTGGG	BGI Genomics	GGH F
GGCTGGAGGAAAGTCTGTCAA	BGI Genomics	GGH R
ATGGAACTGTACCTGGCACG	BGI Genomics	FPGS F
ACCAGGTGGGGAGAGCTAAA	BGI Genomics	FPGS R
GATGTCCGACTTATTCGAGAGC	Sangon Biotech	LC3B F
TTGAGCTGTAAGCGCCTTCTA	Sangon Biotech	LC3B R
GACTACGACTTGTGTAGCGTC	Sangon Biotech	P62 F
AGTGTCCGTGTTTCACCTTCC	Sangon Biotech	P62 R
TTGAAACCTGAAAATGTCCTGCT	Sangon Biotech	AMPKα F
GGTGAGCCACAACTTGTTCTT	Sangon Biotech	AMPKα R
TGTCGGTGGGTATGGACAC	Sangon Biotech	LKB1 F
CTTGCCGTAAGAGCCTTCC	Sangon Biotech	LKB1 R
CGGTCGCAAGCTGTCTCTG	Sangon Biotech	CAMKK2 F
GCGTCCGTTCATGTCCAGG	Sangon Biotech	CAMKK2 R
AGCCCACTTGGATCAGTTTGA	Sangon Biotech	PKA F

GTTCCCGGTCTCCTTGTGT	Sangon Biotech	PKA R
GGCAAGTTCGAGTTCTCCCG	Sangon Biotech	ULK1 F
CGACCTCCAAATCGTGCTTCT	Sangon Biotech	ULK1 R
TGGGGAGTTCAAGGATGCAC	Sangon Biotech	ATG101 F
ACCTTGACCGTCCACACTTC	Sangon Biotech	ATG101 R
TTGCTATAACTAGGGTGACACCA	Sangon Biotech	ATG13 F
CCCAACACGAACTGTCTGGA	Sangon Biotech	ATG13 R
ATCGAAGAGTGTGTACCTACAGT	Sangon Biotech	FIP200 F
GCAGGTGGACGATCACATAAGA T	Sangon Biotech	FIP200 R
CCTGGAAGACCCAATGTTGAAG	Sangon Biotech	PIK3C3 F
CGGGACCATACACATCCCAT	Sangon Biotech	PIK3C3 R
GGTGTCTCTCGCAGATTCATC	Sangon Biotech	BECN1 F
TCAGTCTTCGGCTGAGGTTCT	Sangon Biotech	BECN1 R
TGCACAGAATTGTCTACCTTTCC	Sangon Biotech	PIK3R4 F
GCACATACTGCCTAAAGAGCATA	Sangon Biotech	PIK3R4 R
GTGAAATTACGCCGTCTTGAAC	Sangon Biotech	STX17 F
GATATTGGATCGGAGTTGCTGAA	Sangon Biotech	STX17 R
GCAAATCTTCGACGATCCCAT	Sangon Biotech	ATG14 F
GCAAATCTTCGACGATCCCAT	Sangon Biotech	ATG14 R
TGCACAGAATTGTCTACCTTTCC	Sangon Biotech	PIK3R4 F
GCACATACTGCCTAAAGAGCATA	Sangon Biotech	PIK3R4 R
TCCGAGAGATGAGTCAAGAGG	Sangon Biotech	mTOR F
CACCTTCCACTCCTATGAGGC	Sangon Biotech	mTOR R
TTTGAGCTACTTCGGGTACTTGG	Sangon Biotech	p70s6k F
CGATGAAGGGATGCTTTACTTCC	Sangon Biotech	p70s6k R
**Antibodies**	**Source**	**Identifier**
Gamma glutamyl hydrolase Rabbit mAb (Western blot)	Abclonal	A23578
Anti-GGH antibody produced in rabbit (Immunohistochemistry)	Sigma-Aldrich	HPA025226
ULK1 (D8H5) Rabbit mAb	Cell Signaling Technology	8054s
Phospho-ULK1 (Ser555) (D1H4) Rabbit mAb	Cell Signaling Technology	5869s
LC3B (D11) XP®Rabbit mAb	Cell Signaling Technology	3868s
SQSTM1/p62 (D5E2) Rabbit mAb	Cell Signaling Technology	8025s
FIP200 (D10D11) Rabbit mAb	Cell Signaling Technology	12436s
PI3 Kinase Class III(D9A5) Rabbit mAb	Cell Signaling Technology	4263s

Atg14(D1A1N) Rabbit mAb	Cell Signaling Technology	96752s
Beclin-1 (D40C5) Rabbit mAb	Cell Signaling Technology	3495s
Syntaxin 17 (D3D7H) Rabbit mAb	Cell Signaling Technology	31261s
Phospho-Syntaxin 17 (Ser202) Antibody	Cell Signaling Technology	28630s
AMPKα (D63G4) Rabbit mAb	Cell Signaling Technology	5832s
p-AMPK (THR172) Rabbit mAb	Cell Signaling Technology	2535s
LKB1 (27D10) Rabbit mAb	Cell Signaling Technology	3050s
Phospho-LKB1 (Ser428) (C67A3) Rabbit mAb	Cell Signaling Technology	3482s
CaMKK2 (D8D4D) Rabbit mAb	Cell Signaling Technology	16810s
Phospho-CaMKK2 (Ser511) Antibody	Cell Signaling Technology	12818s
PKA C-α (D38C6) Rabbit mAb	Cell Signaling Technology	5842s
Phospho-PKA C (Thr197) (D45D3) Rabbit mAb	Cell Signaling Technology	5661s
mTOR (7C10) Rabbit mAb	Cell Signaling Technology	2983s
Phospho-mTOR (Ser2448) (D9C2) XP Rabbit mAb	Cell Signaling Technology	5536s
p70 S6 Kinase (49D7) Rabbit mAb	Cell Signaling Technology	2708s
Phospho-p70 S6 Kinase (Thr389) (108D2) Rabbit mAb	Cell Signaling Technology	9234s
LC3B Recombinant Rabbit Monoclonal Antibody (Immunohistochemistry)	Huabio	ET1701-65
SQSTM1 / p62 Mouse Monoclonal Antibody (Immunohistochemistry)	Huabio	EM0704
GAPDH (D16H11) XP® Rabbit mAb	Cell Signaling Technology	5174s
**Regents**	**Source**	**Identifier**
DMEM	VivaCell	C3113-0500
RPMI 1640	Gibco	C11875500BT
Fetal bovine serum	Gibco	10270-106
Penicillin-streptomycin	Gibco	15140-122

Phosphate Buffered Saline	Gibco	C10010500BT
RIPA buffer	Cell Signaling Technology	9806S
PMSF	Beyotime	ST506-2
Protease & phosphatase inhibitor	Thermo Fisher Scientific	1861280
RNA Lipofectamine RNAi max	Invitrogen	13778-150
Opti-MEM medium	Gibco	31985-062
Trypsin-EDTA (1X)	Gibco	25200-056
SDS-PAGE gel	GenScript	M00654
FastPure Cell/Tissue Total RNA Isolation Kit V2	Vazyme	RC112-01
HiScript III RT SuperMix for qPCR (+gDNA wiper)	Vazyme	R323-01
Taq Pro Universal SYBR qPCR Master Mix	Vazyme	Q712-02
TBS-T buffer (10x)	Boster Biological Technology	AR0195-10
Prestained Protein Marker II (10-200 kDa）	Servicebio	G2058
Prestained Protein Ladder	Gene Tech	R1001-002
Crystal violet	Beyotime	C0121
BSA	Sigma-Aldrich	9048468
TB Green Premix EX Taq II	Takara	RR820L
PVDF Western Blotting Membranes	Roche	3010040001
PierceTM BCA Protein Assay kit	Thermo Fisher Scientific	23225
Cell Counting Kit-8 (CCK-8)	Dojindo	CK04
FastPure Cell/Tissue Total RNA Isolation Kit V2	Vazyme	RC112-01
HiScript III RT SuperMix for qPCR (+gDNA wiper)	Vazyme	R323-01
Bafilomycin A1	MedChemExpress	HY-100558
ATP / ADP detection kit	Sigma-Aldrich	MAK135-1KT
NADH	Beyotime	ST358
NADH / $NADH^{+}$ detection kit	Beyotime	S0175
FA/VB9(Folic Acid/Vitamin B9) ELISA Kit	Elabscience	E-EL-0009
AMP-Glo™ Assay	Promega	v5011
ATP Content Assay Kit	Solarbio	BC0305
Glutamic Acid(Glu) Content Assay Kit	Solarbio	BC1585
**Experimental models: Cell lines**		

H838	Cell Bank of the Chinese Academy of Sciences	CC0224
A549	Cell Bank of the Chinese Academy of Sciences	CC0202
H1975	Cell Bank of the Chinese Academy of Sciences	CC0206
MDA-MB-231	Cell Bank of the Chinese Academy of Sciences	TCHu227
**Software and algorithms**		
Image J	Image J	https://imagej.nih.gov/ij/download.html
GraphPad prism 9	GraphPad	https://www.graphpad.com/scientific-software/prism/
R.4.2.0	R	https://www.r-project.org/
